# Survey questions about party competence: Insights from cognitive interviews^☆^

**DOI:** 10.1016/j.electstud.2013.09.005

**Published:** 2014-06

**Authors:** Markus Wagner, Eva Zeglovits

**Affiliations:** Department of Methods in the Social Sciences, University of Vienna, Rathausstraße 19/1/9, 1010 Vienna, Austria

**Keywords:** Cognitive interviews, Party competence, Position, Salience, Survey response, Valence

## Abstract

Voter assessments of party competence have become a key explanation of electoral decision-making. However, there are at least three important aspects to understanding responses to questions on issue-specific party competence: comprehension difficulties; a lack of well-formed attitudes and relevant information; and the use of response heuristics. We used 20 cognitive interviews carried out in Austria in 2011 to test competence questions. The interviews show us how respondents explain their responses. We find evidence that many people (1) may hold only weak opinions and have little information on issue-specific party competence and (2) may make use of distinct but related concepts, particularly salience and position, when answering questions about competence. We provide recommendations for researchers and survey designers based on our findings.

## Highlights

• We study how people answer questions about party competence.
• Important aspects are comprehension, retrieval/judgment, and heuristic use.
• We use results from 20 cognitive interviews conducted in Austria.
• People often lack strong attitudes and useful information on issue competence.
• Individuals may often use salience or position as a response heuristic.

## 1 Introduction

Voter perceptions of party competence on specific issues have become an important way of explaining electoral choices and election outcomes. Voters are said to assess a party's or politician's ability to handle or deal effectively with a political issue (Belluci, 2006; Green and Jennings, 2012a; Walgrave et al., 2012). Those parties and politicians deemed to be particularly competent are then endorsed and rewarded in the voting booth (e.g., Stokes, 1963; Fiorina, 1981; Mondak, 1995; Fournier et al., 2003; Belluci, 2006; Green and Jennings, 2012a,b; Lenz, 2012).^2^ Competence is also part of how parties compete: over time, parties develop reputations for competence on certain issues, and such parties have been said to ‘own’ and campaign particularly heavily those issues (Budge and Farlie, 1983; Petrocik, 1996; van der Brug, 2004; Belanger and Meguid, 2008; Green and Hobolt, 2008; Clark, 2009; Walgrave et al., 2012).

Given the perceived importance of competence to vote choice and party competition, measuring voter assessments of party competence on key issues has become commonplace in election surveys. For example, the most recent surveys carried out by the American, British, Canadian, European and Irish election studies all include detailed questions on perceived issue-specific party competence. However, we know very little about how people answer these questions. When asked to assess the competence of a party or a politician on a specific issue, how do participants in a survey arrive at a response?

Based on existing research on individual attitudes and survey response strategies, we suggest that there are at least three important aspects to understanding responses to questions on issue-specific party competence. Each of these may influence measurement quality as they might affect the responses recorded in the survey.

First, differences in responses may arise not from differences in opinion but from differences in *comprehension*, so what respondents think the terms used in the question mean (Tourangeau, 1984; Tourangeau and Rasinski, 1988; Tourangeau et al., 2009). Second, respondents may lack *well-formed attitudes or relevant information* on issue-specific party competence (Converse, 1964; Krosnick, 1988; Zaller and Feldman, 1992; Chong, 1993; Green and Jennings, 2012a). Yet, they may still give a response to the question (Converse, 1964), often by using satisficing strategies, that is, by giving a response that is good enough rather than as accurate as possible (Krosnick, 1991). Third, responses to competence questions may as a result be characterized by the use of *heuristics*, which are one type of satisficing strategy. Three particular useful heuristics for issue-specific competence are: competence on other issues (Green and Jennings, 2012a); the importance of the issue to the party and the position it takes; and party attachment in general (Rahn et al., 1994).

Identifying measurement issues regarding opinions on issue-specific party competence is important because we need to know what considerations and opinions these responses are likely to reflect. An awareness of potential problems will also help researchers to improve questions on party competence in future surveys. Moreover, problems with the measurement of competence may affect the validity of conclusions based on analyses that make use of these questions (Goerres and Prinzen, 2012). For example, if answers to party competence questions are formulated based on general party affect, then we must be careful in claiming a unidirectional causal link from competence to vote choice (Johns, 2010; Evans and Chzhen, 2013).

In this paper, we use the results from 20 cognitive interviews used to pre-test a series of competence questions for the Austrian National Election Study (AUTNES). We find that some voters only have weakly pre-formed attitudes and limited information on issue-specific party competence and make use of related concepts such as salience and position to provide an answer. In the conclusion, we suggest some potential ways of addressing these measurement issues in issue-specific party competence questions.

We begin by describing in detail the potential ways that voters can answer questions on issue-specific competence. Next, we present the cognitive interviewing method. Then, we assess the results of our research and conclude with recommendations for researchers and survey designers.

## 2 Answering questions on issue-specific party competence

Questions about issue-specific party competence can be seen as questions about attitudes, and research into survey response has theorized that answering such questions proceeds in four steps: comprehension, retrieval, judgment and response (Tourangeau, 1984; Tourangeau and Rasinski, 1988; Tourangeau et al., 2009).^3^ Our study focuses on the first three steps of this process.

### 2.1 Comprehension

First, respondents may differ in what they understand by the words used in the question. Surveys use different words to get respondents to provide issue-specific competence assessments. For example, they are asked which party is best at ‘handling’ an issue (British Election Study) or at ‘dealing with’ a problem (American National Election Study). Personal definitions of these words and terms may vary across respondents. If this is the case, this introduces potential measurement error into voter assessments of competence. As a result, we cannot be sure if differences in responses are due to differences in opinion or just differences in understanding.

### 2.2 Retrieval and judgment

After understanding the question, respondents need to arrive at a judgment regarding issue-specific competence. The memory model and the on-line model of information processing provide two different perspectives on how this occurs. The memory model suggests that respondents store information in their memory; this includes various kinds of considerations, beliefs, feelings or impressions (Hastie and Park, 1986; Zaller and Feldman, 1992). To answer attitude questions, respondents then have to retrieve this information from their memory. Retrieval also means evaluation and selection: the more appropriate and accessible a consideration is, the more likely it will be used to provide a response (Zaller and Feldman, 1992; Tourangeau et al., 2009). A respondent's judgment is then how she combines these considerations in order to reach an overall conclusion. This also means that responses may lack stability if respondents have multiple considerations on the issue (Zaller and Feldman, 1992; Tourangeau et al., 2009: p. 181). For example, Chong (1993) used cognitive interviews to show that, on the issue of civil liberties, people first gave top-of-the-head answers if they were not particularly familiar with the issue. However, they often changed their opinion after thinking about the question at greater length (see also Hochschild, 1981). In sum, in the memory model respondents need not have a pre-formed opinion on issue-specific party competence, but they do need to have some information from which to sample in order to form such a judgment.

An alternative view of the response process is provided by the on-line model of information processing. Here, overall evaluations are formed and updated at the very instant when new information is obtained, and it is this judgment, and not the information and considerations, that is stored in the long-term memory (e.g. Lodge et al., 1989; McGraw et al., 1990). According to the on-line model, respondents would have a pre-formed judgment on issue-specific party competence, even if they may not be able to pinpoint the exact information on which they based this judgment. While the two models of information processing are quite distinct, more recent research reveals that both processes are used by individuals, for example when they evaluate candidates (Redlawsk, 2001; Kim and Garrett, 2012).

So, respondents may provide answers to issue-specific competence questions either based on a pre-formed attitude or based on information stored in their memory. Yet, some or even many respondents have neither such an attitude nor such information (Converse, 1964; Zaller and Feldman, 1992; Fabrigar et al., 2005), especially given the detailed nature of issue-based competence assessments. Moreover, the information respondents have concerning competence may be either (1) not immediately accessible and effortful to retrieve, or (2) complex or even contradictory. Faced with such demands, respondents may reach for satisficing strategies.

Satisficing strategies are present when survey participants merely give a satisfactory answer and not the best one (Krosnick, 1991). In other words, they choose to answer a question that satisfies the interviewer, but one that does not entail careful consideration and judgment. For example, many respondents may give a response instead of saying that they don't know or have no opinion (Converse, 1964). Satisficing may be especially attractive when voters lack well-formed attitudes *and* relevant information, as the question will then be quite demanding for survey participants. However, satisficing is a strategy that is also attractive if information is merely difficult to retrieve or contradictory (Tourangeau et al., 2009: p. 178). So, survey participants may give responses to difficult competence questions by using satisficing strategies. One such strategy is to make use of heuristics.

### 2.3 Heuristics

Three particular heuristics may be used by voters to provide an answer to competence questions: *general party competence*, *related issue assessments* and *party affect*.

First, respondents may use their evaluation of *general party competence* to guide their opinion about specific party competence on other issues (Green and Jennings, 2012a: p. 315). This general competence evaluation is based on the issues that are salient to voters or on which the party has a strong reputation. While many voters are quite likely to have formed some general evaluation of party competence, they often do not ‘possess information to evaluate government or potential party performance across [specific] areas of policy’ (Green and Jennings, 2012a: p. 315f.). In answering questions about specific policy areas, voters may therefore make use of their more general assessments. The consequence of this will be that there is consistency across issues in how voters assess the competence of political parties.^4^

Second, voters may also make use *related issue assessments* in assessing issue competence. Thus, survey respondents may use information from related but distinct concepts to provide an answer to a question. For example, studies in marketing show that consumers often use price as a heuristic or cue for the quality of the product (Leavitt, 1954; Rao and Monroe, 1989). Similarly, voters may make use of other concepts in order to provide an opinion on competence. The issue voting literature highlights two related assessments: issue position and issue salience. That is, voters can also have some belief regarding how important the issue is to the party (RePass, 1971; Budge and Farlie, 1983; Krosnick, 1988; Belanger and Meguid, 2008) and some perception of the party's position (Downs, 1957; Rabinowitz and Macdonald, 1989).

Issue salience can be a useful heuristic for voters as it may indicate whether a party is committed to the policy area and willing to put a lot of its resources into addressing it.^5^ Moreover, respondents could reasonably conclude that a party will rationally decide to address topics more if it is competent on them. If salience is used as a heuristic, then voter assessments of competence may actually reflect their assessment of the salience of an issue to a party.

Party positions are also likely to be linked to how parties are assessed in terms of competence. This is because it is clearly possible to think about competence in terms of positions. For example, a voter may be asked which party would be best at handling the country's economy. If that voter believes that what the economy really needs is another stimulus package, she may regard politicians and parties who instead advocate further cuts in spending as incompetent, even though the disagreement is at least partly positional. Voters are unlikely to believe that parties advocating what they see as the ‘wrong’ solutions are competent actors. For example, a recent study showed that assessments of whether a fictional book author with strong academic credentials was a ‘knowledgeable and trustworthy expert’ were conditional on whether that author shared the respondent's views on climate change, gun control or nuclear waste disposal (Kahan et al., 2011).

Whether positions are linked to competence assessments may depend on the nature of the issue, particularly on the extent of party polarization. Positions are likely to be less influential when all parties agree, so on pure valence issues (Stokes, 1963). There, positions are not a useful guide for assessing competence.^6^ However, if there is disagreement among parties, then positions are more helpful as a heuristic.^7^

In sum, we see party position and issue salience as potential heuristics for competence. As our discussion indicates, one could also see position and salience as legitimate information useful in assessing competence. Yet, even if this is the case, it is still important to know what type of information respondents use to assess competence, which is often treated as distinct from position and salience (e.g. Lenz, 2012).

The final relevant heuristic is *party affect*. Campbell et al. (1960) famously argued that partisanship acts as a ‘perceptual screen’ through which we perceive the world. At the very least, party attachment colors how we respond to survey questions (Rahn et al., 1994), though there is evidence that partisanship shapes our beliefs and actions even beyond the survey context (Gerber and Huber, 2010). In other words, competence and vote preferences may be associated, but not because the former shapes the latter (Johns, 2010, 2011; Evans and Chzhen, 2013). Instead, our broader party preference may influence our beliefs about party competence or, more simply, how we answer competence questions.

## 3 Method: cognitive interviewing

We use information from cognitive interviews in order to examine how people answer issue-specific competence questions. Cognitive interviewing is one of many methods used in testing questionnaires (e.g. Presser et al., 2004; Campanelli, 2008), and its usefulness arises from its ability to shed light on the cognitive processes during survey response.

To document these processes, two methods are widely used in cognitive interviews: thinking aloud and verbal probing (Biemer and Lyberg, 2003; Willis, 2005; Beatty and Willis, 2007). The think aloud technique encourages respondents to verbalize their thoughts, so they are asked to say out loud whatever they are thinking of while trying to find an answer to a question. In contrast, verbal probing relies on explicitly formulated additional questions that aim at gaining more insight into the respondents' thought processes. For example, a respondent may be asked why she gave a particular answer or what she takes certain words in the question to mean. The think aloud technique has been found to be particularly useful in understanding the process of information retrieval, while it is better to use verbal probing to gather information on comprehension (Willis, 2004). This is why we used both techniques in our cognitive interviews.

We carried out 20 cognitive interviews between July and September 2011 to test survey questions for the Austrian National Election Study (AUTNES) 2013. In the beginning of the interview, respondents were trained to use the think aloud technique following the pre-test-procedure used for the ANES 2006 (DeBell et al., 2009).

Later in these interviews, we asked four groups of questions about issue-specific competence. These questions were chosen based on the kinds of questions asked in previous academic surveys. Some questions ask about past party performance: for example, the American National Election Study asks whether respondents approve or disapprove of the way the federal government handled the war in Iraq, the war in Afghanistan and terrorism. Other surveys ask about hypothetical (or future) party competence: for instance, the British Election Study asks how well respondents think a government formed by the current opposition would handle specific issues. Finally, respondents can be asked which party is generally most competent at dealing with an issue. An example is the popular question inquiring which party would be best at handling the issue or solving problem identified as the most important by the respondent. So, we asked questions concerning past, hypothetical and general issue-specific performance. We present the questions we tested in Table 1.

We split the questions into two pre-test questionnaires so as to keep the length of the cognitive interviews to a manageable length of less than one hour. We asked Group A the standard question on which party would be best at handling the issue that the respondent identified as being the most important. We asked Group B three types of questions about party competence on two specific issues, immigration and education. For each topic, we asked which party is the most and which is least competent (general issue-specific competence); which party did the best and which did the worst job since the last election (past performance); and which party was likely to do the best job in the coming years and which was likely to do the worst (future/hypothetical performance).

In Austria, the issues of immigration and education differ in several ways. First, immigration is more salient to voters than education, as evidenced by the electoral success of the populist radical right. Second, Austrian parties are strongly polarized on immigration, with the social-democratic SPÖ and especially the Greens taking a more pro-immigration stance. On education, it is mainly the two governing parties, the SPÖ and the Christian-Democratic ÖVP, who have clear positions. Between these two parties, polarization on this issue is also significant as they disagree on matters such as tuition fees and the structure of the school system. Finally, immigration can be classed as an ‘easier’ issue than education: as a political topic, immigration is more symbolic and less technical than education, and it deals more with policy ends than with means (Carmines and Stimson, 1980). In sum, immigration is a highly salient, highly polarized and ‘easy’ issue, while education is moderately salient, moderately polarized and ‘hard’.

We recruited participants from all over Austria, making sure to include in our sample people with lower levels of education and a low interest in politics, as they are more likely to lack well-formed attitudes (Zaller and Feldman, 1992) and use satisficing strategies (Krosnick, 1991). Recruiting strategies included announcements in supermarkets and job centers as well as contacting target persons within our own social networks. Our final set of interviewees consists of 13 women and 7 men, aged between 17 and 76, of different educational and social backgrounds (see Table 2). Interviewees were given 20 Euro for the interview. Interviews were carried out by six different members of the AUTNES team. All interviews were audio-taped and transcribed, and the resulting text is the data for our analysis. All translations were carried out by the researchers.

Since interviewees were not sampled but recruited, we cannot claim representativeness. In general, cognitive interviews mainly tell us whether and what kinds of problems can arise during survey response. This means that our findings cannot be used to conclude anything about the distribution of responses to party competence questions (Goerres and Prinzen, 2012). Moreover, while any problems we uncover may apply only to a small number of people, they are equally likely to apply to a large proportion of potential respondents. So, the interviews tell us what kind of problems may arise, not how often.

The analysis was carried out by the authors. In doing so, we collected responses and other information (e.g. the time lag between questions and answers, answering with a question) that were relevant to the following questions.

• Comprehension: How are the terms used understood (handling an issue, being competent, doing a good or a bad job)? Do they mean to respondents that parties have the ‘ability to deliver results’? If not, what do respondents take the terms to mean?
• Retrieval and judgment:
  ○ How do respondents justify their answers? Do they refer to specific events or actors?
  ○ Are there signs of unstable or weak attitudes and a lack of information, such as admitting a lack of knowledge or struggling to provide an answer? Do respondents struggle to provide a judgment, or do they struggle to justify their judgment?
• Heuristics:
  ○ General party competence: When assigning competence to a party on an issue, do respondents talk about other issues or about the party's general competence?
  ○ Related issue assessments: In giving an answer, do respondents refer to concepts such as salience or position?^8^
  ○ Party affect: Do respondents refer to a general disposition toward the party? Do their answers fit with their overall party sympathies?

## 4 Results

In the following, we present the results for comprehension, justification of answers and heuristics.

### 4.1 Comprehension

In general, the comprehension of the concepts of ‘competence’, ‘handling an issue’ and ‘doing a good/bad job’ was satisfactory. Thus, many interviewees connected these terms with relevant characteristics such as knowledge and ability, so whether a party is generally an expert on the specific topic. Interviewees also linked competence to realism and pragmatism. Parties were seen as competent when they set realistically achievable goals that could actually be implemented, and they were incompetent when they made promises that they knew would be impossible to realize. For ‘competent’, some examples are:

RB4: Competent means for me to have actual knowledge, well-founded knowledge. That means knowing statistics and having personal experience with the issue and not just via newspaper articles […].

RB6: Competence means I have some knowledge and some ability […].

RB7: If I am competent this means that I have the ability to achieve something, to work on it, to talk about it.

RB9: [The FPÖ is least competent on immigration] because the party does not offer or aim for any realistic solutions.

This realism and pragmatism is often akin to a dispassionate, objective analysis of the situation, as in this response given by interviewee number B9.

RB9: Competent means for me: [The party] has a clear analysis of the current situation and how it should be, and a clearly formulated decision on what to do. [The SPÖ is most competent on education] because the party wants to renew our education system and make it fit for the present age.

Some examples for ‘handling’ are:

RA8: To handle an issue, to me, means to collect all possible information, background knowledge, and to respect the position of others.

RA7: To handle it? Yes, to handle it: that they really take care of things and not only talk, that they really do something that will be implemented. That they don't just promise things before the election, but that it is actually implemented.

RA9: [To handle an issue means] that to come to a result you cooperate no matter which party you belong to, and that you come to a result.

An example from ‘doing a good/bad job’ is:

RB4: [Doing a good job means] that they have a realistic idea of what they want to achieve and that they implement this to a certain extent.

Interviewees thus often referred to knowledge, realism and dispassionate analysis, which are all conceptually close to competence. In a general sense, we therefore have evidence that these respondents do have a good understanding of what competence means and what a competent party would look like.

However, some problems nevertheless arose among respondents. Concerning ‘competence’, there were a couple of references to salience:

RB2: Well, [competent on immigration means for me] that they campaign the most on it, that they work hard for immigrants.

RB6^9^: [I think], that the Greens for example deal with it most frequently and thus are most competent.

Some respondents also linked ‘handling an issue’ to electoral success.

RA1: [to handle an issue means] that the party is able to take the chance to win voters here.

RA5: To handle an issue, in principle, means how you sell yourself in the end. […] on election day, that the votes are there again.

In sum, although many interviewees had an understanding of competence that is at least similar to the theoretical concept, some clearly confused the concept with that of salience, while some had a completely different understanding of competence. Below, we will see that references to position and salience were even more frequent when respondents were asked to explain their choice of the most competent party.

### 4.2 Retrieval and judgment

How do interviewees arrive at the judgment they give, and what information do they use? To ascertain this, we use responses to our probings, so how interviewees explained their answers to us. While we cannot distinguish between on-line and memory-based information processing in our research design, we can nevertheless gain some insight into how respondents answer issue-specific party competence questions. We also wanted to find out more about how difficult it is for respondents to answer these questions, so whether they have a judgment or at least information at their immediate disposal. We thus considered whether respondents struggled in giving an answer. In the analysis, we identified struggling if respondents did one or more of three things: they explicitly stated that they had difficulties; they needed time to give an answer; and if they answered our question with a question of their own.

#### 4.2.1 Question difficulty and response speed

Interviewees generally found the issue-specific competence questions rather difficult. In many cases, there were long pauses before interviewees answered the question. In several cases, interviewees even freely owned up to having little knowledge about the matter:

RA9: [On which party is best at handling the most important issue:] I can't say that right now at all.

RB1: [On the best job on education:] Since the national parliamentary election? Well, now you can…that's very difficult. It's…I feel not much has changed.

RB5: [On education competence:] I haven't paid enough attention recently [to answer that].

RB7: [On the worst job on immigration] Worst? I don't know, no.

If these respondents choose the ‘don't know’ option in a survey, this lack of attitudes would not induce a measurement error. However, in our interviews some other respondents did not admit that they had difficulties. Instead, they answered with a question, or the hesitated for a couple of seconds before they answered.

RB3: [Least competent on education:] (hesitates) The Greens or the BZÖ? I don't know now. Interviewer: From your point of view. There is no right or wrong answer. RB3: Yes, yes. So let's say the BZÖ.

RB7: [who did the worst job on education]: On the topic of education, the worst? (Pause.) The topic of education, I don't know if you can do a bad job there. I just think that may be the Greens were least concerned by the topic or maybe it wasn't one of their main topics.

While some interviewees struggled with all competence questions, not all questions were equally difficult. Interviewees generally tended to have a much easier time assessing immigration competence than education competence. This applies in particular to the question of who is *least* competent on immigration: five of our ten respondents picked the radical-right FPÖ, and did so without much hesitation. Another three chose the Greens, again with little time needed to reflect on the answer. To us, this indicates that answering competence questions is less difficult on ‘easy’ and highly salient topics where there is strong polarization between parties. This could provide support for Chong's (1993: p. 897) finding that ‘top-of-the-head answers can be highly reliable if they pertain to commonly discussed subjects with established frames of reference’. Yet, as we discuss below, these quick responses may also stem from the use of related issue assessments or general party affect. Moreover, detailed responses reveal that some interviewees managed to name a party and did so quickly, but that they cannot explain why.

RA2: [explaining why she chose SPÖ as most competent on the most important problem]: That just occurred to me. I don't have a particular reason [for answering that].

RB3: [On the best job on education:] The SPÖ and the ÖVP, or should I just name one. Interviewer: Yes, just one. RB3: The ÖVP. Interviewer: Why? RB3: I'm not sure exactly, but I can only remember, that I praised them sometime because of education. This is a bit embarrassing for me now. I really can't remember right now, but I think that's still the best answer.

Such answers could be evidence of on-line information processing, but given the difficulties these interviewees had providing any justification at all, it might be more likely that they made use of satisficing strategies, for instance heuristics, to provide an answer. We therefore now turn to the question of the factors interviewees referred to as justifications for their answers.

#### 4.2.2 Justifying answers: information sources

Respondents only rarely referred to specific events or actors as a reason for their response. There were nevertheless a couple of exceptions. One respondent (RB4) remembered a specific event. To justify why the Greens were most competent on education, she referred to a specific situation where she learned about the Greens' views at an event organized by them. The same respondent did not choose the Greens as the party who did the best work in the past four years because in her opinion they had failed to prevent a certain bill being passed in parliament. Another respondent (RB10) referred to the work of the ministers of education when talking about party competence. Interestingly, she did not use the same mechanism for immigration.

RB10: [justifying why no party is most competent for education] I have experienced education ministers from various parties… None of them reinvented the wheel.

Many interviewees referred to the media as an important source of information, no matter whether they paid a lot of attention to politics or not. A couple of examples are:

RB3: [On education competence:] I don't really have much of an idea, but I think that is right given what little I read in the newspaper.

RB8: [On education competence:] Well there I have to go through all the parties and look at education…and I can only answer it in relation to what I hear subjectively in the media and because I don't know the party program. … So when I say competent, then it's their impression on me, that they leave a positive impression.

So, interviewees often reported vague impressions based on general media reporting rather than carefully considered judgments or views based on specific events or actions.

In addition to the source of the information cited, we were also interested in the nature of this information. In particular, we considered the use of heuristics to guide the response.

### 4.3 Heuristics

#### 4.3.1 General party competence

In our interviews, we found only little evidence that interviewees made use of general competence assessments in determining their response. One example was the following:

RB10: The FPÖ [is least competent on education] – but you can put that as my answer for everything, that party isn't competent on anything.

However, this was an exception, and this answer perhaps also reflects general party affect. Moreover, interviewees did not explicitly refer to other issue areas in explaining and justifying their answer. They also did not tend to drift off into the discussion of other topics when doing so. Of course, we cannot exclude that respondents were not aware that they were transferring other competence assessments to the issue at hand, but at least this is not a strategy that our interviewees appeared to engage in consciously.

#### 4.3.2 Related issue assessments

In contrast, there is strong evidence that interviewees made use of related issue assessments. Many interviews clearly show that respondents assess competence in ways that mix relevant characteristics with assessments of salience and position. Some examples on salience are as follows:

RB4: [on immigration:] Because [the Greens] just campaign the most on it. So on asylum matters…it was a lot mainly from the Greens. They advertised a lot and invested a lot of time.

RB7: The FPÖ [is the least competent on education] I think, because somehow I think they're not interested in it at all.

RA10: [on party best at handling most important issue:] I think, [the SPÖ] have a stronger commitment than the ÖVP. I do not know about the other parties, because you do not hear a lot from them.

Importantly, all three respondents cited above had a valid comprehension of competence, that is, they defined it as having the status of an expert in terms of ability or knowledge. Here, we thus arguably have particularly strong evidence that the use of salience is not a matter of comprehension but of satisficing.

Some respondents also referred to positions, though this occurred less frequently. Again, we limit ourselves to those who gave a valid definition of competence:

RB4: [on education:] … I got this flyer and I read it carefully and I liked a lot what [the Greens] were offering there and what they were campaigning for. … So I thought of the Greens, because that's the last thing that stuck in my mind.

RB8: Well, the Greens always combine the issue [of immigration] with the economy, and that approach really appeals to me.

Overall, respondent assessments of competence are often based on more than just expertise and the ability to get things done. Instead, they justify their answer based on the fact that a party was very visible on the topic or campaigned on it. They also on occasion linked competence with making good, realistic suggestions that seem like the right solutions. Such statements have clear policy content as well. Importantly, we are not arguing here that respondents are giving ‘wrong’ answers. As we state above, position, salience and competence are difficult to disentangle, so it is no surprise that voters do not do so. However, it is important for people who use competence questions to be aware that other, related assessments clearly feed into the way people arrive at answers. Indeed, we would argue that the use of such assessments can amount to a satisficing strategy.

#### 4.3.3 Party affect

Finally, there are some occasions where party affect influenced responses. Thus, some interviewees framed their answers explicitly in reaction to their general party identification.

RA9: I can't say [which party is best at dealing with the Euro crisis]. I am very disappointed by the ÖVP. I am actually an ÖVP voter since I can remember. I was a convinced ÖVP voter. But for ten years I am no longer that, but just out of habit.

Interviewer: And why would you say that [the SPÖ is the most competent party on immigration]? RB9: The SPÖ? Now, not just because I just declared: I am a Social Democrat.

As with the general party competence heuristic, the influence of party affect may be difficult to detect since respondents are unlikely to acknowledge openly that their partisan leanings influence their answers. Moreover, respondents need not necessarily be aware of this influence in the first place. Nevertheless, these comments by our interviewees do show that party affect may on occasion shape how people assess issue-specific competence.

Our interviews also shed light on general party sympathies, which we also list in Table 2. Respondents with strong partisan leaning are amongst the ones who had difficulties with the questions, but named a party that was most competent without being able to explain why they did so. One example was RA3:

RA3: [who is most competent on the most important problem, which is Greece]: (hesitates): Oh (Pause.) Instinctively I'd answer – I would say the SPÖ still.

RB1, who leaned strongly towards the SPÖ, chose SPÖ as most competent for the issue of immigration without hesitating. However, he did not say that other parties were not competent, and gave no explanation about what makes the SPÖ competent.

Moreover, we could examine the extent to which party competence assessments matched this overall affect. In general, interviewees gave responses that matched their partisan leaning, especially if their affect was strong. In those cases, interviewees did not tend to contradict their overall loyalties. Among some partisans, however, there were interviewees who were open to thinking about other parties as being competent. For example, the SPÖ partisan RB1 mentioned above chose the Greens as most competent for education, but in his explanation he defended the SPÖ:

RB1: [The SPÖ] is tied to the ÖVP, they simply cannot… But they do not want tuition fees, either… [SPÖ chancellor] Faymann did not say anything meaningful.

Partisan leanings therefore play a strong role in how interviewees think about and judge issue-specific party competence, even if they choose a different party as their response.

### 4.4 Response options

Finally, in the course of our analysis we became aware of another difficulty concerning competence questions: assigning judgments to response options. In our case, interviewees were asked to name one party as the most or least competent. However, in some cases, responses indicate that more than one party can be seen as the most competent. A single answer option may unduly narrow down voter beliefs. A few examples are:

RB7: [On immigration competence:] I think that the two coalition partners, so the SPÖ and the ÖVP, I think that they can hopefully find a very good solution and – the most competent, I think I can't choose just one… Interviewer: And if you had to choose just one. RB7: Just one…I don't know.

RA2: [On which party is best at handling the most important issue:] I would say the SPÖ and the ÖVP. Interviewer: Ok, and if you were only able to give one party? RA2: Then I would probably say the SPÖ.

When asked about the party that had done the best job since the last election, interviewees thus almost exclusively concentrated on the two government parties (SPÖ and ÖVP). To the extent that voters have opinions about competence, some question formats may encourage respondents to consider mainly the larger and/or governing parties.

RB3: [On education:] I can take the SPÖ or the ÖVP…since the last election?

RB7: [On education:] …the SPÖ is more for staying down if you get a 5 [i.e. low grades], from what I have heard and I don't think that's so good, so that means I would take the ÖVP then.

RB8: [On education:] I can't think of anything related to the Greens or the FPÖ and education. I only know that I am not satisfied with the ÖVP's education policy.

The matter of response options is therefore complex. On the one hand, asking about competence may encourage respondents to think about governing parties in particular. In coalition systems, respondents may therefore select among parties currently or recently in government rather than considering the whole range of parties. On the other hand, some respondents may find it difficult to choose just one party as the most competent. This would suggest that a rating rather than a ranking scale may be more appropriate for competence questions.

## 5 Conclusions and recommendations

Questions about issue-specific party competence are generally difficult and demanding. While our findings on comprehension are encouraging, responses to issue-specific party competence questions appear to be characterized by a lack of information and pre-formed judgments. Voters often only have weak prior opinions on the competence of particular parties on specific policy fields, and the information necessary to from judgments during the interview itself is often either unavailable or difficult to retrieve. It is likely that information on issue-specific competence is simply hard for voters to acquire. For example, opinions seemed particularly weak on the less salient and ‘hard’ issue of education, where it was also difficult for interviewees to assess whether opposition parties had ‘done a good job’. The consequence of weak attitudes and a lack of information is that respondents may give a response to a question, but not one that is based on previously held opinions or information. This means that responses to these questions may lack reliability.

Instead of being based on an attitude or information, the answers given often reflect the use of related assessments as heuristics, so people use information on the issue position and emphasis of actors as a guide to responses to competence questions. Many respondents appeared to deduce competence from the amount of time a party talks about a topic and how much it advertises these elements of its program. It is very difficult to separate competence, salience and position even theoretically, so it is perhaps not surprising that interviewees also failed to do so. In any case, it is important for researchers to be aware that measures of competence may often reflect perceptions of other issue characteristics, namely salience or position. Crucially, this may be the case even on ‘easier’ issues such as immigration. Overall, this means that issue-specific party competence questions may lack validity.

In our interviews, the use of a general party competence heuristic and of a party affect heuristic appeared to be less frequent. However, we cannot conclude that these problems do not occur. For one, party preferences strongly influence competence assessments (Evans and Chzhen, 2013), so perhaps our interviews simply show that respondents are not actively aware of using such cognitive shortcuts. Moreover, research by Johns (2010) and particularly by Green and Jennings (2012a) has shown that general party competence is perhaps a better way of understanding the role of competence assessments in vote choice. Green and Jennings (2012a) thus provide a useful account of how specific issue assessments may feed into and shape perceptions of general party competence.

Finally, we also have five suggestions for survey designers who wish to measure issue-specific party competence.

1. Express the concept in terms that respondents are likely to understand. Referring directly to ‘competence’ may be more useful than terms that allow respondents to interpret the question in terms of position or salience (e.g., ‘deal with an issue’).
2. Use rating scales or allow for multiple responses on competence questions as voters sometimes believe that more than one party is competent on an issue.
3. Include issue-specific questions not just on competence, but also on salience and position. These measures could then be used as tight statistical controls in order to provide stringent tests for the effect of competence assessments. However, to separate out analytically each type of issue assessment more sophisticated research designs such as panel data may be necessary (Evans and Chzhen, 2013; Sanders et al., 2011).
4. Separate positional, salience and competence questions as well as party preference questions within the survey. Question proximity may prime certain considerations among respondents, particularly if earlier questions can act also act as attractive heuristics (Lau and Sears, 1983; Zaller, 1992; Bartels, 2002; Evans and Chzhen, 2013).
5. Ask follow-up questions about voters' competence assessments. Respondents are particularly likely to have only weak prior opinions and knowledge on issues they know little about. It would be useful to record this in the survey itself, for instance, by asking respondents how certain they are about their assessment or how important that policy area is to them (Fabrigar et al., 2005: p. 26). Another possible option is to give respondents the explicit option of saying that they do not know or have no opinion regarding party competence on an issue.

To conclude, our findings do not mean that issue competence is not an important factor in explaining electoral choices. However, our interviews do show that researchers need to be careful when interpreting these questions, and that care needs to be taken when designing and analyzing surveys that aim to measure issue-specific party competence.

## Figures and Tables

**Table 1:** Questions asked in cognitive interviews.

	Question asked	Verbal probing	Questionnaire
1	Which party is best at handling the issue [most important issue given by respondent in previous question]?	What do you understand by “handling an issue best”?	A

2	Introduction: independently of whether you agree with a party's position or not, how competent are the parties in handling an issue? 2A. Which party is most competent for the issue of [education, immigration]? 2B. Which party is least competent for the issue of [education, immigration]?	2A. What do you understand by a party being “competent”? 2B. Why did you choose party [X] as the most competent party? 2C. Why did you choose party [Y] as the least competent party?	B

3	Introduction: Thinking of the time since the last federal election, 3A. Which party did the best job in the area of [education, immigration]? 3B. Which party did the worst job?	3A. What do you understand by a party “doing a good or bad job”? 3B. Why did you choose party [X] as having done the best job? 3C. Why did you choose party [Y] as having done the worst job?	B

4	Introduction: And thinking of the coming years, 4A. Which party will do the best job in the area of [education, immigration]? 4B. Which party will do the worst job?		B

**Table 2:** Participants in the cognitive interviews.

Questionnaire	Respondent	Gender	Age	Occupation	Region	Partisan leaning	Political attentiveness & sophistication
A	RA1	Male	37	Sports coach	Vienna	Weak: SPÖ	Rather high
A	RA2	Female	38	Employee	Vienna	Supports SPÖ/Green coalition	Moderate
A	RA3	Female	50	n/a	Vienna	Strong: SPÖ	Very attentive, but moderate knowledge
A	RA4	Male	50	n/a	Tyrol	Supports SPÖ/FPÖ coalition	Moderate
A	RA5	Male	n/a	n/a	Tyrol	If at all: SPÖ	High
A	RA6	Male	41	Unemployed	Tyrol	No	High
A	RA7	Female	ca. 40–50	Waitress	Salzburg	Weak: ÖVP	Low
A	RA8	Male	45	Driver	Vienna	No	Moderate
A	RA9	Female	76	Retired	Vienna	Strong: ÖVP	High
A	RA10	Female	30	n/a	Vienna	Weak, if at all: SPÖ	Low
B	RB1	Male	ca. 45	Concierge	Vienna	SPÖ	Very attentive, but moderate knowledge
B	RB2	Female	17	High school student	Vienna	Prefers Green/SPÖ	High
B	RB3	Female	75	Retired	Lower Austria	If at all: SPÖ	Very low
B	RB4	Female	24	Student	Vienna	Supports Green positions	Very high
B	RB5	Female	53	n/a	Tyrol	No	Low
B	RB6	Female	ca. 40	Unemployed	Tyrol	No	Moderate
B	RB7	Female	17	In education (catering)	Lower Austria	No	Moderate
B	RB8	Female	35	Self-employed IT expert	Vienna	Prefers Green/SPÖ	Rather high
B	RB9	Male	60	Medical doctor	Vienna	Strong: SPÖ	High
B	RB10	Female	65	Retired	Vienna	Strong against FPÖ	High

## Footnotes

2 This paper focuses on *issue-specific* competence assessments of political parties. We do not consider overall competence assessments of political parties (e.g. Green and Jennings, 2012a) or of candidates (e.g. Mondak, 1995; Besley, 2005), which measure voters' summary judgments of parties or candidates.

3 Biemer and Lyberg (2003) argue that there are five stages, as they include record formation or encoding as the first step preceding comprehension.

4 The problem of such ‘issue transfer’ only applies to issue-specific assessments of party competence and not to general assessments.

5 Walgrave et al. (2012) call this *associative* issue ownership, which they contrast with *competence* issue ownership.

6 However, we note that Sanders et al. (2011) argue that positional considerations on a spatial issue can be used a competence heuristic for general valence issues. Our argument is different in that we believe it may be possible for positional views to influence competence perceptions *on the same issue*.

7 Some issues do not have a level of abstraction at which most citizens agree. For example, there is fundamental disagreement on desirable goals on issues such as abortion or immigration. The use of positions as a heuristic may be particularly likely here, as there is no valence aspect to an issue.

8 When respondents mix competence with related concepts such as salience or position, it is not always straightforward to decide whether this was due to a different comprehension of competence or a satisficing strategy. We addressed this as follows in the analysis: (1) we interpreted references to salience and position as indicators of problems in comprehension if they occurred when respondents were specifically asked for their comprehension of the key terms; (2) we interpreted references to salience and position as indicators of the use of related issue assessments if respondents used such references when explaining why they had chosen a specific party, in particular if they had provided a valid definition of the concept before.

9 The same respondent defines competence differently at another place of the interview, as ‘trying somehow to change things through initiatives’.
