# Corrigendum to “Survey questions about party competence: Insights from cognitive interviews” [Elect. Stud. 34 (2014) 280–290]

**DOI:** 10.1016/j.electstud.2014.06.007

**Published:** 2014-09

**Authors:** Markus Wagner, Eva Zeglovits

**Affiliations:** Department of Methods in the Social Sciences, University of Vienna, Rathausstraße 19/1/9, 1010 Vienna, Austria

The authors wish to add the following acknowledgement to the article:

“This research was conducted under the auspices of the Austrian National Election Study (AUTNES), a National Research Network (NFN) sponsored by the Austrian Science Fund (FWF) (S10902-G11)”.

